# Detection of Serotype-Specific Antibodies to the Four Dengue Viruses Using an Immune Complex Binding (ICB) ELISA

**DOI:** 10.1371/journal.pntd.0002580

**Published:** 2013-12-26

**Authors:** Petra Emmerich, Angela Mika, Herbert Schmitz

**Affiliations:** 1 Department of Virology, Bernhard Nocht Institute for Tropical Medicine, Hamburg, Germany; 2 Diagnostics Development Group, Bernhard Nocht Institute for Tropical Medicine, Hamburg, Germany; University of North Carolina at Chapel Hill, United States of America

## Abstract

**Background:**

Dengue virus (DENV) infections are preferentially diagnosed by detection of specific IgM antibodies, DENV NS1 antigen assays or by amplification of viral RNA in serum samples of the patients. The type-specific immunity to the four worldwide circulating DENV serotypes can be determined by neutralization assays. An alternative to the complicated neutralization assays would be helpful to study the serotype-specific immune response in people in DENV hyperendemic areas but also in subjects upon DENV vaccination.

**Methods:**

In consecutive samples of patients with DENV-1- 4 infection type-specific antibodies were detected using an immune complex binding (ICB) ELISA. During incubation of serum samples and enzyme- labeled recombinant envelope domain III (EDIII) antigens immune complexes (ICs) are formed, which are simultaneously bound to a solid phase coated with an Fc–receptor (CD32). After a single washing procedure the bound labeled ICs can be determined. To further improve type-specific reactions high concentrations of competing heterologous unlabeled ED III proteins were added to the labeled antigens.

**Results:**

Follow-up serum samples of 64 patients with RT-PCR confirmed primary DENV-1, -2, -3 or -4 infections were tested against four enzyme-labeled recombinant DENV EDIII antigens. Antibodies to the EDIII antigens were found in 55 patients (sensitivity 86%). A complete agreement between the serotype detected by PCR in early samples and the serotype-specific antibody in later samples was found. Type-specific anti-EDIII antibodies were first detected 9–20 days after onset of the disease. In 21% of the samples collected from people in Vietnam secondary infections with antibodies to two serotypes could be identified.

**Conclusions:**

The data obtained with the ICB-ELISA show that after primary DENV infection the corresponding type-specific antibodies are detected in almost all samples collected at least two weeks after onset of the disease. The method will be of value to determine the distribution of the various type-specific anti–DENV antibodies in DENV endemic areas.

## Author Summary

Infections with four different dengue viruses are threatening 2.5 billion people in tropical countries. Since most antibodies to these four viruses are cross-reacting, a type-specific ELISA would be valuable to study the immune response to the circulating viruses in patients but also in healthy subjects in endemic counties. Therefore a novel DENV immune complex binding (ICB) ELISA was developed to detect serotype-specific antibodies to all four dengue virus serotypes in human serum samples. The tests use labeled recombinant EDIII antigens of the four DENV strains. Numerous samples of patients with RT-PCR confirmed dengue fever were assessed by the new method. In samples of 55 patients with primary dengue fever full agreement between the serotype detected by RT-PCR and the serotype-specific antibody based on the ICB ELISA was obtained. The type-specific antibodies were not observed before the second week of illness. Our data suggest that using the ICB ELISA in healthy adult subjects in an endemic region (Vietnam) both primary and secondary infections can be identified. The method may help to analyze the distribution of the four dengue viruses in the tropics.

## Introduction

Dengue fever is a highly prevalent arthropod-borne viral disease with 2.5 billion people in tropical or subtropical areas at risk for infection. The clinical picture of dengue may vary considerably from mere fever to severe shock syndrome. The annual number of infections is estimated to several hundred million [1], [2].

As four DENV serotypes exist, humans can be exposed to DENV infections several times. While dengue fever is usually associated with a rather low mortality, dengue hemorrhagic fever may give rise to serious and sometime lethal complications. It has been shown by several studies that dengue hemorrhagic fever is frequently but not always due to secondary DENV infection [3]–[5]. Therefore the detection of serotype-specific IgG antibodies would be of value to determine the immunological anti-DENV profile of an individual but also of a larger population in endemic countries. Knowing the serotype-specific antibody response, the risk of secondary infections with a new serotype can be predicted. Information on serotype-specific antibodies may also help to monitor the immune response after successful DENV vaccination [6], [7].

Early after onset of acute DENV infection the serotype involved can be detected by RT-PCR [8]–[11], or by NS1 antigen detection [12], [13]. However, several weeks after onset of infection both methods will no longer give positive results. In contrast, even years after human infection, serotype-specific IgG antibodies can be detected by the plaque reduction neutralization test (PRNT). However, up to several months after primary and even more after secondary infection subtype cross-reactivities are observed by PRNT [14], [15]. Moreover, the PRNT is both time consuming and also difficult to handle, because the four different DENV strains have to be propagated in a BSL2 laboratory [16] and due to various technical details a standardization may be difficult to achieve [14], [17].

Meanwhile it has been shown for many flaviviruses that upon acute infection type-specific antibodies to the domain III of the viral envelope (EDIII) are produced. EDIII is regarded as putative receptor binding domain [18], [19], and strong neutralizing antibodies against individual flaviviruses localize to EDIII [20], [21]. Monoclonal antibodies binding to the EDIII of DENV envelope glycoprotein are the most efficient inhibitors of virus adsorption to Vero cells [20], whereas monoclonal antibodies that cross-react with other flaviviruses localize primarily to the envelope domain II [22]–[24]. A humanized monoclonal antibody to loop A of DENV-2 EDIII was able to protect mice from lethal infection [25]. However natural variation of the DENV genotypes may influence the binding of neutralizing antibodies to EDIII proteins [26]. In addition neutralization is not confined to the EDIII [24], [27], [28]. Of 40 monoclonal antibodies recognizing DENV-2- infected cells, 14 bound to yeast cells displaying the DENV-2 EDIII domain. Most of these antibodies directed to the envelope EDIII domain are serotype-specific, but minor DENV cross-reacting and flavivirus cross-reacting epitopes were also identified on the EDIII protein [24], [29]–[32].

Due to the low homology with other flaviviruses the EDIII domain of DENV has been frequently used as antigen to increase the specificity of DENV IgG [27], [33], [34] The anti-EDIII assays were considerably more specific than assays using larger DENV antigens or complete virions but had a sensitivity of only 80% in detecting acute DENV infections, which may be explained by the small number of epitopes on the EDIII protein [23], [35]. A detailed analysis of the EDIII type-specific immune response both in primary and secondary DENV infections has been presented by Midgley et al. 2011 [36]. They used an indirect ELISA with the four EDIII antigens coated on solid phase. The inhibition was carried out by pre- incubating different dilutions of the serum samples with different dilutions of the four EDIII antigens. Without inhibition 25 samples of children with confirmed DENV-1, -2 and -3 primary infection were studied. In two patients detailed results of the competitive assay were presented. 17+10 samples of children with DENV-1-4 secondary infection were also analyzed both with a competitive ELISA and with PRNT. While with primary infections the type-specific results were in agreement with PCR subtyping, in secondary infections only the memory response to the preceding DENV infection was found, supporting the theory of the “antigenic sin”. Recently, homodimeric EDIII proteins were applied for the discrimination of serotype-specific DENV IgM antibodies, but only IgM antibodies of DENV-1 infected patients showed clear subtype-specific reactivity [37].

All these investigations show that EDIII proteins may also be of diagnostic significance for the detection of serotype-specific antibodies to DENV. Since our earlier immunoblot technique to detect anti-DENV EDIII activity in serum samples of patients with DENV infection [38] could not differentiate between type-specific and cross-reacting anti-EDIII antibodies, we developed a more sensitive and even more specific immune-complex binding (ICB) ELISA, which has already been shown to detect antibodies to recombinant WNV and tick-borne encephalitis virus (TBEV) EDIII antigens with high specificity and sensitivity [39], [40]. The ICB assay is controlled by two specific immunological mechanisms: first the antibodies to be determined react with the horseradish peroxidase (POD)-labeled antigen in the wells of microtiter plates and simultaneously the aggregated antibodies attach to an appropriate Fc-receptor such as FcγRIIa (CD32) coated onto the solid-phase plates [41].

As an advantage of the ICB ELISA, POD-labeled and unlabeled, closely related antigens can be combined to competitively suppress unwanted cross-reacting antibody activities. Thus, by combining TBEV POD- labeled EDIII antigen with an excess of unlabeled WNV EDIII antigen, highly specific antibody assays could be established [40], [41]. A similar combination of POD-labeled and unlabeled DENV EDIII antigens was introduced here, to establish epitope-specific antibody assays. As we had collected numerous consecutive serum samples of patients, for whom the DENV serotype had been determined by RT-PCR, we were able to assess the anti-EDIII response in DENV infected patients and to evaluate the serotype-specificity of the four ICB ELISAs by using the respective recombinant DENV EDIII proteins as antigens.

In addition, we studied the distribution of DENV type-specific antibodies in serum samples of healthy subjects from Vietnam, a hyper-endemic area with many secondary infections [42], [43].

## Materials and Methods

### Ethics statement

From all patients in this study informed written consent for the detection of antibodies had been obtained prior to processing the samples. In minor age patients (<18 years) informed consent was given on his or her behalf by a parent. The collection of serum samples of patients with dengue fever was approved by the Ethics Committee of the Ärztekammer Hamburg (WF-024/11). All data on human subjects were analyzed anonymously. Clinical investigations have been conducted according to the principles expressed in the Declaration of Helsinki. Studies on sera from healthy Vietnamese people had been approved by the Scientific Council of Education, Training and Ethics of Hué Medical School on September 11, 1998 [44].

### Serum samples

During the last ten years consecutive samples of 64 European tourists with acute dengue fever (20–55 years of age m/f: 1.2) had been collected during our routine diagnostics [31]. All samples had been aliquoted and stored at −20°C. In early samples no antibodies to DENV were detected, while the DENV serotype could be identified using a real time RT-PCR protocol [9]. According to the PCR results 25, 15, 17 and 7 sera were obtained from DENV-1, DENV-2, DENV-3 and DENV-4 infected patients, respectively. Subsequent serum samples taken at least one week after onset of fever were available from all 64 patients. In all secondary samples IgM and IgG antibodies to DENV were demonstrated by indirect immunofluorescence (IIF) using virus infected Vero cells [38]. In addition in 2012, 5 mL serum per patient could be obtained from seven patients with PCR confirmed DENV infection during the convalescent phase. Thus sufficient material was available for DENV neutralization assays.

12 samples of patients with WNV infection (8 North Americans and 4 Germans), 5 samples of Japanese encephalitis virus (JEV) vaccinees, 11 of yellow fever virus (YFV) vaccinees and 20 samples (10 clinical cases and 10 vaccinees) of subjects with antibodies to TBEV were derived from our routine diagnostics. The presence of antibodies to the respective viruses had been confirmed using our highly specific ELISAs [39], [41] and IIF.

To test the specificity of the DENV ICB ELISA serum samples of 88 subjects of our routine diagnostics (25–59 years, m/f: 1.5) were included. The samples diluted 1∶10 in phosphate buffered saline (PBS) had no antibodies to DENV as shown by IIF.

In addition, 87 serum samples of healthy Vietnamese people (18–65 years m/f: 1.5) had been obtained during studies on amoebiasis and dengue fever at the city of Hue in 1999. 71 samples contained antibodies to DENV as shown by IIF (titer 1∶>20).

### DENV RT-PCR

For the detection of the four DENV serotypes in early serum samples of infected patients a real time RT-PCR protocol with four primer-probe sets in a single reaction mixture was applied [9]. The RT-PCR assays were run on a LightCycler 480 System (Roche, Mannheim, Germany). A mixture of all four DENV RNAs served as positive and inhibition controls.

### PRNT

DENV micro-neutralization [14], [17], [27], [31] was performed in Vero E6 monolayers grown in 96-well plates. The cells were infected with the same DENV strains that had been used for cloning and expression of the ED3 proteins. DENV immune sera were heat- inactivated, serially diluted in twofold steps (1∶20 to 1∶10,240) in nutrition medium and the dilutions were preincubated in triplicate with 50 plaque forming units determined for each DENV strain in a final volume of 100 µl for 1 h at 37°C. The serum/virus mixtures were added to the cells for 1 h at 37°C. Then the wells were emptied and filled with cell culture medium (MEM; 2% FCS) to a total volume of 250 µl containing 0.75% methylcellulose. After incubation for 96 h at 37°C in 5% CO_2_ the cells were washed, fixed with 5% formalin in PBS and permeabilized with 0.5%Triton ×100 in PBS. The cells were immunostained with a cross-flavi mouse monoclonal antibody (2F1), followed by POD-labeled anti-mouse antibody and precipitating TMB. Dilutions were plotted against spot counts in triplicate and 50% plaque reduction neutralization was calculated by nonlinear dose–response regression analysis (Prism 6 Package, GraphPad Software, Inc., San Diego, CA).

### Expression and purification of DENV EDIII antigens in *E. coli*

For the production of the four recombinant DENV EDIII proteins the following dengue strains were used: DENV-1 West Pac, Genbank Accession–No. U88535; DENV-2 New Guinea C, Genbank-Accession-No. AF038403; DENV-3, H87 Genbank-Accession-No. M93130; DENV-4 Thai1978, Genbank-Accession-No. U18441.The four EDIII antigens of DENV-1-4 (aa 297–400) of the E protein were cloned in expression vector *pQE30* with an N-terminal His- tag [38]. Upon IPTG-induced expression of the His-tag fusion proteins, the transformed cells (*E.coli* JM109, Promega, Mannheim, Germany) were dissolved in 8M urea and the EDIII proteins purified by nickel affinity chromatography as described earlier [38]. The antigens were eluted using a pH gradient. After renewed binding to fresh Ni–NTA and matrix- associated refolding a second elution with PBS containing 0.25 M imidazole, 5% glycerol was performed. The material was stored in 20% glycerol at −20°C. Purity of refolded antigens (about 0.4–0.8 mg/mL) was controlled by SDS-PAGE and Coomassie staining.

### Expression and purification of CD32 in *E.coli*

The extracellular part of CD32 (FcgRIIa H131) [45] was cloned into expression vector pJC45 [46] using amplification with the primers 5′ACGCATATGGGACTTGAAGTCCTCTTTCAGGGACCCGGGCAAGCTGCA GCTCCCCCAAAG-3′ and 5′-ACCGGAATTCTTAGATCCCCATTGGTGAAGAGC-3′ and restriction enzymes NdeI and EcoRI, respectively. After heat shock transformation in *E. coli* BL21 (*pAPlacIQ*), IPTG-induced expression of the His-tag fusion protein was performed at 18°C over night. Cells were lysed by lysozyme and sonification [47] (modified). Washed inclusion bodies were solubilized in 6M guanidine hydrochloride, 50 mM Tris-HCl, pH 7.8 and the recombinant protein was purified by Ni-NTA under denaturing conditions. Protein fractions were combined, refolded by rapid dilution and concentrated in centrifugal filters. The His-tag was cleaved off by digestion with 3C protease (GE Healthcare, Stockholm, Sweden) at 4°C over night. To remove the protease and cleaved off tags further purification was performed using gel filtration chromatography with an ÄKTA pure FPLC system (GE Healthcare, Stockholm, Sweden). Purity was visualized by SDS-PAGE with silver staining and with Western blotting using an anti-human CD32 polyclonal antibody (R&D Systems, Minneapolis, USA).

Purified CD32 was bound to microtiter plates (Immunolon, Nunc, Wiesbaden, Germany) at a concentration of 5 µg/mL in PBS containing 1 mg/mL NaN_3_ and was incubated on the plates for at least three days at 4°C before use. Sealed microtiter plates could be stored at 4°C for two months or at −20°C in the presence of 25% glycerol for at least one year.

### Labeling of recombinant DENV EDIII antigens

The antigens were directly labeled with POD as described earlier [39]. 4 mg POD type IV (Sigma Aldrich, Taufkirchen, Germany) in 0.5 mL distilled water were activated using 0.1 mL 0.1 M sodium periodate. After dialysis against 1 mM acetic acid buffer (pH 4) 1 mg of recombinant DENV EDIII antigen were added at pH 9.3. The mixture was threefold concentrated. After incubation at 4°C over night, the antigens were diluted 1∶10 in PBS containing 1% BSA, 1% fetal calf serum and 20% glycerol. Addition of NaBH_4_ was not necessary. The POD-labeled, 1∶10 diluted DENV-1, -2, -3 and -4 EDIII antigens (DeP1-4) could be stored at −20°C for at least two years without any loss of activity. All DeP1-4 antigens were applied in the ICB ELISA at a dilution of 1∶≥16,000 (1–2 nM of antigen) using a diluent containing 1% BSA, 1% fetal calf serum and 1% detergent (Triton ×100; Sigma Aldrich, Taufkirchen, Germany) in PBS.

### Competitive addition of related flavivirus antigens

To increase the serotype-specific reactions, *i.e.* to eliminate antibody cross-reactions due to cross-reacting epitopes on all DePs, an excess of heterologous, unlabeled DENV EDIII antigens (De1-4) was added to the labeled DeP1-4. Using the ICB ELISA with DeP1, DEP2, DeP3 and DeP4 as antigens and the respective positive control sera the amount of unlabeled De 1,2,3 and 4 antigens for complete autologous inhibition was determined. A 100-fold excess of unlabeled to labeled antigen was usually sufficient to suppress the reaction with the positive control sera. Besides, using varying concentrations of the respective autologous De1, 2, 3 and 4 antigens the inhibitory strength of newly produced De batches could be quantified and compared. Similarly, a 100-fold excess of De3 added to DeP1, 2, 4 (w/w) and of De1 added to DeP3 antigen was able to competitively block subtype-DENV cross-reactivity. For instance the addition of a final dilution of 1∶160 of De3 (1 mg/mL) to the DeP1 (containing 1 mg/mL De) diluted 1∶16000 was sufficient to obtain complete inhibition of subtype reactivity.

Moreover, to avoid false positive reactions due to antibodies to the His-tag, to POD or *E.coli* proteins, POD-labeled EDIII TBEV antigen (TBEP) [40] was added to the DePs. The competitive addition of the labeled antigen required the irreversible inactivation of the enzymatic activity of POD. To this end 1 mL of the labeled TBEP was incubated with 0.1 mg NaN_3_ and 0.035% H_2_O_2_ [48] for at least three days at 4°C followed by dialysis over night against PBS containing 10% glycerol. The inactivated antigen (iTBEP) did no longer show enzymatic activity at a dilution of 1∶100 (no reaction after 10 minutes with TMB substrate solution). The antigenicity of iTBEP was unaffected by the NaN_3_ treatment, as was documented by competitively adding iTBEP to TBEP using the TBEV ICB ELISA [41]. A complete inhibition of the reaction of the positive control was seen in the presence of iTBEP up to a dilution of 1∶400.

### Anti-DENV ICB ELISA

The ICB ELISAs were performed as described earlier for the detection of antibodies to the EDIII antigens of WNV and TBEV [41]. Prior to testing the microtiter plates coated with CD32 were rinsed three times with washing buffer (100 mM Tris, 150 mM NaCl, 0.05% Tween 20). Protein blocking of the plates was not required. Human sera were diluted 1∶10 in PBS containing 0.5 mL/L ProCline 300 (Bellafonte, PA, USA) and 10 mg/L phenol red for better visualization. In each assay a positive, and two negative control serum samples were included to monitor interassay variation. To control for false positive results, one of the negative samples contained anti-HIS-tag antibodies, which are frequently found in sera of patients with falciparum malaria [40]. Aliquots of positive control sera for each of the four assays were stored at a dilution of 1∶20 at −20°C. Each 25 µl of diluted serum and 25 µl of the labeled ED3 antigen (DeP; diluted 1∶≥16000) were added to the wells of the microtiter plate and the mixture was incubated at 4°C over night. Finally, plates were washed three times with the washing buffer and stained with 50 µl TMB (KPL, Gaithersburg, MD USA) per well at room temperature for 10 min. After adding 100 µl 1N sulphuric acid optical density (OD) was read at 450/620 nm.

### Statistics

For each of the four ICB ELISAs the cut-off values (mean OD+3σ) were calculated using the 88 negative samples.

In addition, for each ICB ELISA accuracy, optimum sensitivity and specificity were determined using the Two Graph Receiver Operating Characteristic (TG-ROC) curve analysis (MedCalc statistical software B-8400 Ostend, Belgium). The OD values of 110 negative samples (from 88 blood donors and from 22 subjects of Table 1) served as true negatives (criterion 0) and the OD signals of the 25+15+17+7 RT- PCR positive samples of the tourists as true positives (Criterion 1).

**Table 1:** False positive reactions obtained with any of the four DENV ICB ELISAs and with IIF.

Serum donors	Number	ICB ELISA	ICB ELISA + iTBEP	Dengue IIF
DENV negative controls	88	5	0	0
WN patients	12	0	0	12
TBEV vaccinees	20	0	0	7
JEV vaccinees	5	0	0	3
YFV vaccinees	11	0	0	4
Total	**136**	**5**	**0**	**26**

∶>10. ICB ELISA positive: P/N. 136 serum samples without antibody to DENV were compared. In the absence of competitive iTBEP antigen the ICB ELISA produced 5 false positive results. IIF positive: 1

The results of the ICB ELISA are presented as positive/negative (P/N) ratio by dividing the OD of the sample by the OD of the cut-off value. P/N values≥1 were considered as positive.

Intra- and interassay coefficients of variation (CVs) were calculated for each ICB ELISA ((Prism 6 Package, GraphPad Software, Inc., San Diego, CA) using our four positive control sera in rows of sixfold, tested on three consecutive days.

### Accession numbers

DENV-1 West Pac, Genbank Accession–No. U88535;

DENV-2 New Guinea C, Genbank-Accession-No. AF038403;

DENV-3, H87 Genbank-Accession-No. M93130;

DENV-4 Thai1978, Genbank-Accession-No. U18441

## Results

**Figure 1:**
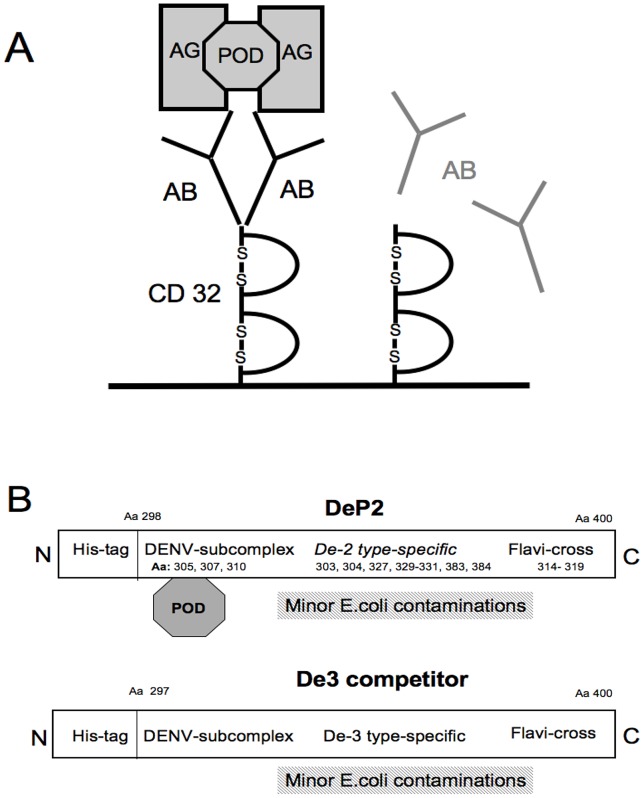
ICB ELISA procedure. **A**: During incubation of serum samples containing antibody (AB) and POD-labeled recombinant antigen (POD, AG) immune complexes form, which in turn are attached to the solid-phase coated with CD32. Non-specific antibodies (in grey) do neither bind to the antigen nor to CD32. Competition with unlabeled antigen is not shown. **B**: Schematic drawing of the POD-labeled EDIII domain of DENV-2 (DeP2) and of the unlabeled EDIII domain of DENV-3 (De3) serving as competitor in the ICB-ELISA. While type-specific epitopes on the DeP2 and De3 are different from each other, cross-reacting epitopes are almost identical. In the presence of an excess of De3, cross-reacting antibodies will no longer bind to sub-domain epitopes on the DeP2 antigen. Amino acids (aa) involved in epitope formation [29] are also indicated. Of note, a subcomplex (aa 305,307) and a type-specific (aa 303,304) epitope are in close proximity.

In this study a type-specific DENV ICB ELISA was applied, which can avoid the binding of non-specific or cross-reacting antibodies by competition between labeled and unlabeled antigens [39], [40]. The principle of the ICB-ELISA is shown in figure 1A. Serum samples together with a labeled antigen are added to microwells coated with the recombinantly produced Fc-receptor (CD32). During incubation immune complexes develop, which simultaneously bind to CD32. After a single washing procedure, the amount of labeled immune complexes on the solid-phase can be measured.

The rationale behind the competition is illustrated in figure 1B. Two DENV EDIII antigens, one POD-labeled and the other one unlabeled are shown. The unlabeled DENV-3 EDIII antigen (De3) competes for antibodies to identical, *i.e.* cross-reacting epitopes on the POD-labeled DENV-2 EDIII antigen (DeP2). Anti-DENV cross-reacting antibodies will almost exclusively bind to the unlabeled De3, provided that the De3 competitor is added in excess to the DeP2 antigen. Thus only antibodies to the type-specific epitopes on the labeled DeP2 will be detected. In addition type-specific adjacent to cross-reacting epitopes (fig. 1B, aa 303–307) may no longer be masked by binding of cross-reacting antibodies.

We started our experiments without competition using chessboard titrations. Positive control sera of four patients, who had PCR–confirmed DENV-1, -2, -3 or -4 infections, were tested against different dilutions of the labeled DENV antigens, designated as DeP1, DeP2, DeP3 and DeP4 (see materials and methods). For better comparability of the data the results of the four ELISAs are presented as P/N ratios. Maximum P/N ratios were obtained using the DeP1, DeP2, DeP3 and DeP4 antigens at rather high dilutions of 1∶≥16 000. Two samples of subjects without DENV infection were always included as negative controls. All sera were tested at a dilution of 1∶10, but some sera were positive up to a dilution of 1∶10 240). Using the OD values of the 88 negative samples the cut-off OD levels for the four DeP1-4 ICB ELISAs (mean OD+3σ) were 0.1, 0.09, 0.1 and 0.16, respectively.

Moreover a pool of broadly cross-reacting serum samples from Vietnamese subjects was tested without competition. The four ELISAs produced an absorbance of 1.8, 1.8, 1.7 and 1.9, respectively. The data suggest that the four ICB ELISAs detect subtype-specific antibodies to the different DeP antigens with similar sensitivity.

Intra-assay and inter-assay CVs for the ICB ELISAs using four of the above mentioned control samples was <10% for the DeP1-4 antigens, when the assays were performed on three consecutive days (see Table S1; supporting information).

### False positive results

Samples from 88 patients, who had never entered DENV endemic areas and did not report any flavivirus vaccinations, were tested using the four DENV EDIII ICB ELISAs and IIF (Table 1). All 88 samples were negative in IIF, but five samples were positive in the four ICB ELISAs (Table 1: third column). These false positive reactions were probably due to antibodies to the His-tags [40], to traces of *E.coli* contaminations or to the POD in the DePs. When all samples were retested using the DeP1-4 antigens in the presence of a 100-fold excess of the iTBEP EDIII antigen (see material and methods), all 88 samples were negative (forth column; + iTBEP. The addition of iTBEP did not significantly alter the reaction of anti-DENV positive serum samples. Therefore all further DENV ICB-ELISAs were performed in the presence of a 100-fold excess of iTBEP antigen.

Although we knew from earlier experiments that using the ICB ELISA with EDIII proteins as antigens flavivirus cross-reacting antibodies rarely occur [41], we tested 12 samples of patients with WNV infection, 20 samples of subjects with anti-TBEV antibodies, five samples of JEV- and 11 samples of YFV vaccinees with both the ICB ELISA and IIF (Table 1). All 48 samples were negative (P/N ratio≤1) with all four DENV serotype-specific ICB ELISAs (specificity 100%). In contrast, using anti-DENV IIF, 26/136 samples (Table 1, last column) were false positive (all 12 anti-WNV samples, 7 sera with anti-TBEV antibodies, 3 sera of JEV vaccinees and 4 samples of YFV vaccinees).

### Sensitivity of the ICB ELISA using the DeP1-4 antigens

Over several years consecutive serum samples of 64 tourists with acute DENV infections had been obtained. All patients had primary infections (no anti-DENV IgG antibodies in the early PCR positive sample [49]). To confirm the DENV type-specific results subsequent samples were available 6–15 days after onset of fever. In 55 of these 64 subsequent samples antibodies to at least one of the four DeP antigens were found (overall sensitivity 86%). However, out of 12 samples taken more than 10 days after the onset of the disease 11 were positive. Obviously, antibodies to EDIII come up late during the course of the disease. However, they seem to persist for prolonged periods, because in two PCR positive patients with DENV-1 and -2 infection, where diagnostic samples were obtained three and six months after onset of infection, the initial antibody reactivity to the DeP antigens had not decreased.

To obtain additional information on the accuracy, sensitivity and specificity of each of the four ELISAs a Two Graph Receiver Operating Characteristic (TG-ROC) analysis was applied (see supporting information, figure S1). The analysis was carried out using the OD values of the 110 negative samples (criterion 0) and the 64 DENV PCR positive samples (criterion 1). The cut-off levels used in this study based on mean OD+3σ of the 88 blood donor samples resulted in sensitivities for the DeP1-4 ICB ELISAs between 80% and 100% and specificities between 94% and 100%. But due to the relatively small number of positive samples a wide range for the 95% confidence limits (Cl) was obtained. The accuracy of all four tests was between excellent and good (ROC>0.85).

### Type-specific reactions and DENV sub-complex reactivity

**Figure 2:**
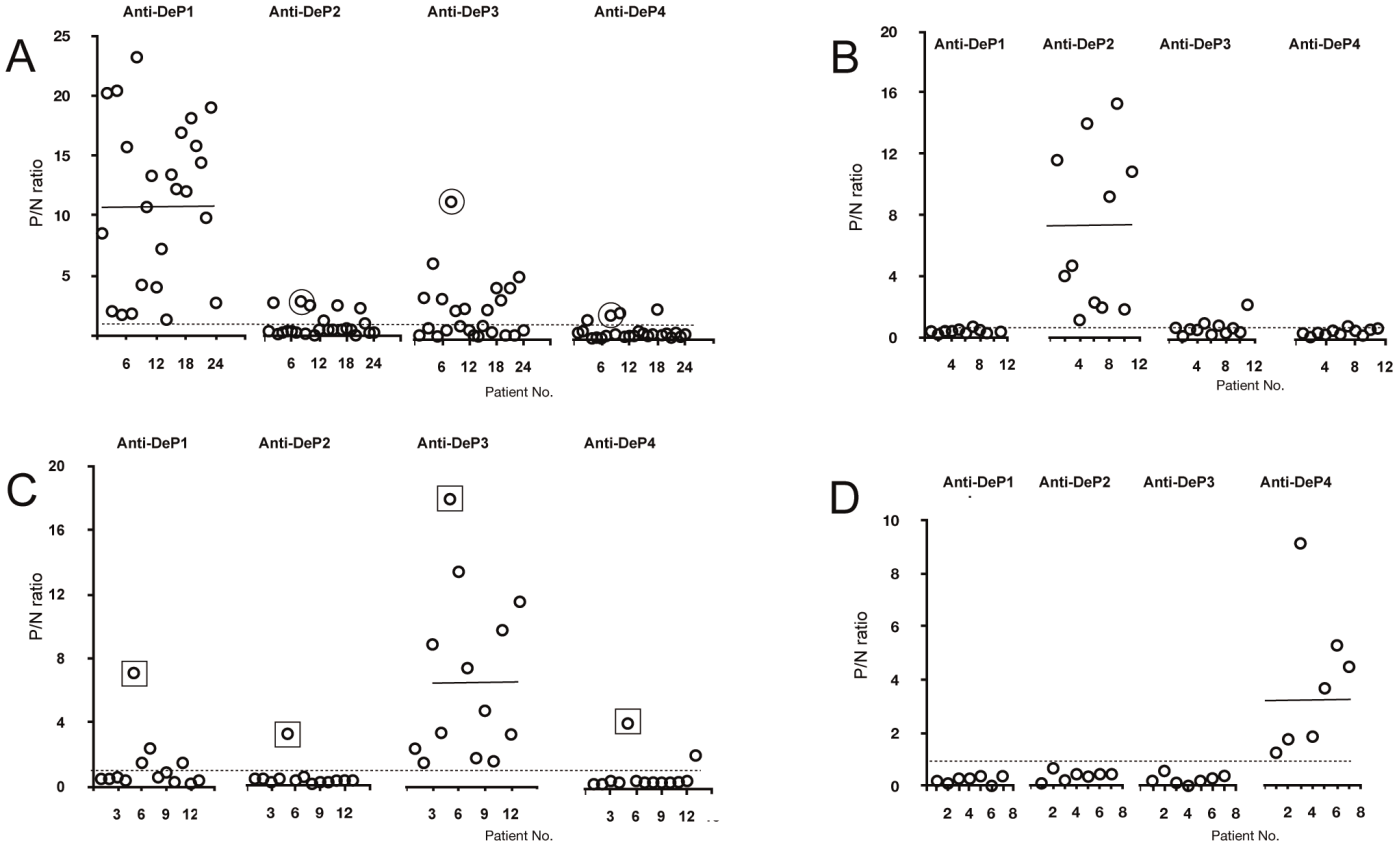
Antibody reactions (P/N ratios) to all four DENV DeP antigens using the ICB ELISA without competition. **A**: Sera of 24 patients with DENV-1 infection. **B**: sera of 11 patients with DENV-2 infection. **C**: sera of 13 patients with DENV-3 infection. **D**: sera of 7 patients with DENV-4 infection. DENV serotype had been confirmed by RT-PCR. Dotted lines represent cut-off values. Sample of patient 8 in A and of patient 5 in C with strong cross-reactivity are marked with circle and square, respectively. P/N values were calculated using OD/cut-off values.

DENV serotype-specific reactions using the 55 anti-DeP positive samples are shown in figure 2A–D. The tests were run without competition by De antigens. 24, 11, 13 and 7 samples were positive with DeP1, 2, 3 and 4 antigens, respectively. The reactivity (P/N ratio) of the 24 samples of PCR-confirmed DENV-1 infected patients with the DPe1 antigen ranged between 1.3 and 23.2 (figure 2A). The majority of the 24 sera did only weakly react with the other DeP antigens. Only in sera with strong anti-DeP1 reactivity DENV major subtype cross-reactions, preferentially with DeP3, were observed. However, the reactivity (P/N ratio) with the homologous DeP antigen exceeded by far the signals observed with heterologous DeP2, 3, or 4 antigens (figure 2A: patient 8; encircled data points). Likewise, in 11 patients with proven DENV-2 infection (figure 2B), in 13 patients with DENV-3 infection (figure 2C; patient 5 with strong cross-reactivity; boxed data points) and in 7 patients with DENV-4 infections (figure 2D) the antibody reactions with homologous DeP clearly exceeded that obtained with the heterologous antigen. Overall, cross-reacting responses were seen in about 25% of the samples. The 220 OD values of the 55 positive samples are shown in Table S2.

### Competitive ICB ELISA

**Figure 3:**
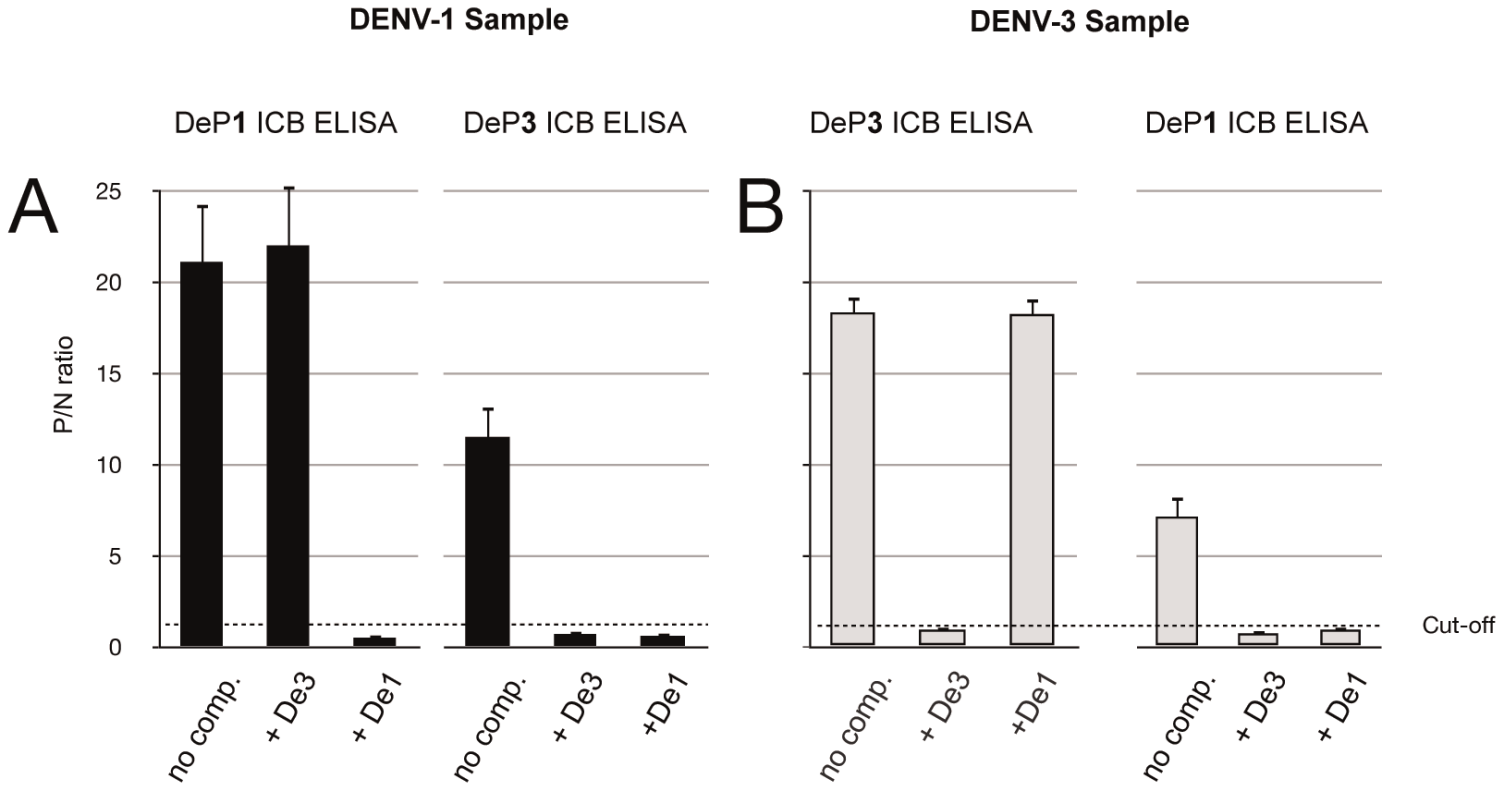
Competitive ICB ELISA using two cross-reacting samples. **A**. Reactivity (P/N ratio) of a DENV-1 sample. Black columns from left to right: without addition of competitive antigen (no comp.) a strong reaction with DeP1 (P/N ratio 21.1) is seen. Next column: Upon competition with De3 (De3+) the reaction with DeP1 (P/N ratio 22) remains almost unchanged. Next column: autologous competition with De1 (+De1) results in a P/N ratio of <1. Cross-reactivity with DeP3 antigen (next three columns): without competition (no comp.) the P/N ratio is 11.5. With competition using De3 (+De3) and De1 (+De1) all reactivity disappears. **B**. The same experiment is carried out for the DENV-3 sample (grey columns). Without competition (no comp.) a strong reaction with DeP3 antigen is seen (P/N ratio 18.5). Addition of De1 (+De1) does not change the positive reaction (P/N ratio from 18.3 to 18.2). The strong cross-reactivity with DeP1 (P/N ratio 7.1) without competition (no comp.) disappears both upon addition of De3 (+De3) and of De1 (+De1).

To further reduce DENV subtype cross-reactions observed with some highly reactive serum samples, competitive ICB ELISAs were performed. Examples of the competitive assays are shown in figure 3A and B using sera of two patients, one with DENV-1 (encircled in Fig. 2A) and the other with DENV-3 infection (boxed in Fig. 2C). Using the DENV-1 positive serum a P/N ratio of 21.1 and a cross-reactivity (P/N ratio 11.5) was seen with DeP1 and DeP3, respectively, without addition of competitors (Fig. 3A; no comp.). Addition of unlabeled De3 protein did not significantly alter the positive signal of the DENV-1 serum with DeP1, but minimized the cross-reactive signal with DeP3. A similar result is shown in figure 3B for an exemplary DENV-3 serum. A complete suppression of the cross-reaction with DeP1 on competition with De1 was observed, while the strong reaction with DeP3 was unchanged. Based on this assay principle the exact amount of competitor needed to completely suppress type- and subtype-specific reactivity could be determined. For most unlabeled antigens an excess of De over DeP of 100-fold (w/w) was sufficient.

**Figure 4:**
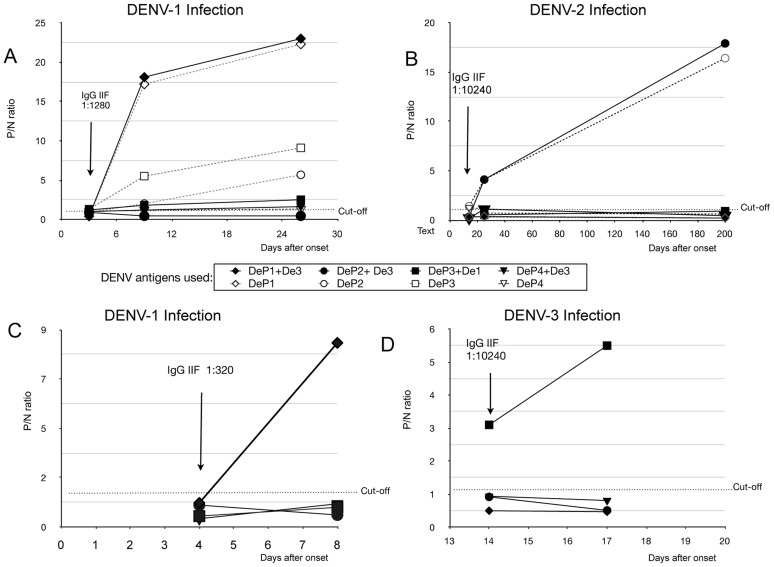
Antibody kinetics of four patients with DENV-1, -2 or -3 infection. **A**. Samples of DENV-1 infected patient collected on days 3, 9 and 26 after onset. **B**. Samples of a DENV-2 infected patient, collected on days 14, 25 and 200. **C. D**. Samples of a DENV-1 and a DENV-3 infected patient. ICB ELISAs were performed with competition (filled symbols) and without competition (open symbols) by De antigens. Initial IIF titers are indicated.

By combining highly diluted, labeled DeP1-4 antigens with low dilutions of unlabeled heterologous EDIII antigens (for example DeP1, DeP2 and DeP4 combined with a 100-fold excess of De 3, and DeP3 with an 100-fold excess De1) all subtype-specific cross-reactions, as shown in figure 2A–C, could effectively be reduced to P/N ratios≤1 without altering the type-specific reactions by more than ±10% (see also figure 4 and supporting information, figure S2). In conclusion, using the ICB ELISA the serotype-specific antibody response in all 55 primary DENV infections was in full agreement with the serotype as determined by RT-PCR.

### Kinetics of type-specific antibodies to DeP antigens

In four patients with PCR confirmed DENV-1, -2 and -3 infection consecutive serum samples could be collected. The antibody response of a DENV-1 infected patient to all four DePs on days 3, 9 and 26 is shown in figure 4 A. The ICB ELISA with competition (filled symbols) and without competition (open symbols) was applied. The strong cross-reactivity with DeP3 (open diamonds) could almost completely be removed by adding De3 to DeP1 (filled diamonds: anti-DeP1+De3), while the addition of heterologous antigen did not reduce but rather enhanced the autologous antibody response to DePs. In a patient with DENV-2 infection (figure 4B) almost no subtype-reactivity with DEP1, 3, 4 was seen. Again, the addition of De3 to DeP2 did slightly increase the type-specific response on day 200. In figure 4C and D antibody reactions of two consecutive samples of another DENV-1 and of a DENV-3 infected patient are presented. As stated before, type-specific antibodies were only detected after the first week after onset of the disease, while in samples taken during the first week high IIF antibody titers to DENV were already present. The OD values used for figure 4 A–D are listed as supporting information in Table S3.

### PRNT assays

Sufficient amounts of serum for PRNT testing could be obtained from seven DENV infected tourists. A comparison of the P/N ratios, IIF titers and PRNT titers of these serum samples are shown in Table 2. Both the PRNT titers and the results of the competitive CB ELISA confirmed the serotype obtained by RT-PCR, but the PRNT showed strong cross-neutralization especially with the DENV-2 samples. The results obtained with the seven samples taken several weeks or months after onset do not support a close correlation between IIF-, PRNT titers and ICB ELISA-derived P/N ratios.

**Table 2:** Comparison of IIF titers, PRNT titers and P/N ratios obtained with RT-PCR confirmed sera of seven patients with primary DENV infection.

Serotype and	Days after onset	IIF Titer	PRNT				Competitive ICB ELISA **P/N ratio (means of 3 testings)**			
patient #			DENV- 1	DENV-2	DENV-3	DENV-4	DeP1+De3	DeP2+De3	DeP3+De1	DeP4+De3
**DENV-1** Sample EM 34	26	1∶640	**1∶707**	1∶<20	1∶132	1∶<20	**23.2**	0.44	1.0	0.8
**DENV-1** Sample EM30	13	1∶320	**1∶718**	1∶24	1∶694	1∶583	**8.2**	0.42	1.0	0.26
**DENV-2** sample EM 9	12	1∶81920	1∶872	1∶1694	1∶936	1∶523	0.33	**2.3**	0.12	0.22
**DENV-2** Sample EM25	80	1∶2560	1∶247	**1∶575**	1∶165	1∶255	0.2	**7.6**	0.72	0.25
**DENV-3** Sample EM35	35	1∶40960	1∶732	1∶1095	**1∶3868**	1∶2200	1.0	0.33	**3.4**	0.18
**DENV-3** sample EM 2	17	1∶10240	1∶203	1∶<20	**1∶3577**	1∶108	0.5	0.21	**11.1**	0.8
**DENV-4** EM 31	36	1∶2560	1∶279	1∶173	1.122	**1∶5462**	0.4	0.3	0.2	**5.1**
Negative sample		1∶<10	1∶<20	1∶<20	1∶<20	1∶<20	0.3	0.2	0.2	0.4

### Serotype-specific antibodies in Vietnam

**Figure 5:**
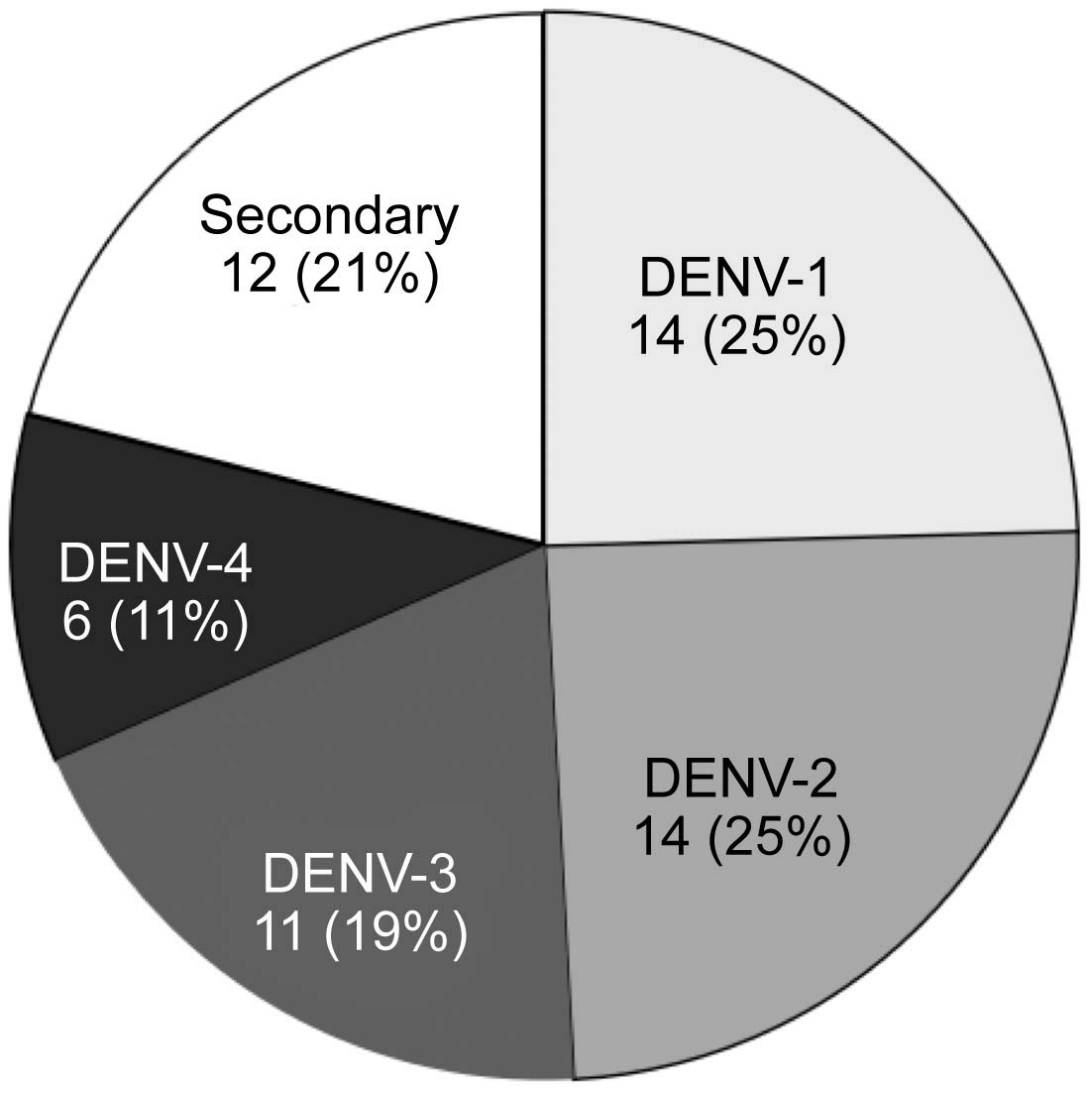
Distribution of DENV serotype-specific antibodies in sera of 57 healthy people from Vietnam. 12 subjects (21%) showed reactions with two DeP antigens.

To learn more about the distribution of DENV serotypes in an endemic region, additional sera of 87 adult healthy Vietnamese people were analyzed using the four DENV ICB ELISAs. No PCR data were available. 71 of the 87 samples (82,6%) were positive by IIF, but false positive reactions due to cross-reactions with other flavivirus antigens could not be excluded. 57 of the 71 IIF-positive samples (80%) reacted with at least one of the four DeP- antigens. Without competition 35 samples showed DENV subtype cross-reactions with three to four DeP antigens. Therefore all 57 samples were retested in the presence of an excess of heterologous antigen, *i.e.* DeP1, DeP2 and DeP4 antigens were combined with a 100-fold excess of De3 while DeP3 was combined with the same excess of De1. Using the competitive version of the ICB ELISA 45 of the 57 subjects (79%) produced positive reactions (P/N ratios>1) with one antigen only. 14, 14, 11 and 6 samples reacted with DeP1, DeP2, DeP3 and DeP4, respectively (figure 5). Therefore the most prevalent serotype- specific antibodies were directed to DENV-1 (DeP1) and DENV-2 (DeP2) followed by DENV-3 (DeP3) and DENV-4 (DeP4). Provided that the detection of two or more DENV type-specific reactions in a serum sample mean secondary infection, 12 subjects (21%) with secondary infections were found. Four reacted with both DeP1 and 3; three with DeP3 and 4; two with DeP1 and 4; three with both DeP1 and 2. To exclude residual cross-reactions the 12 samples were also tested at a serum dilution of 1∶100. The P/N ratios obtained with all four antigens are shown in Table S3. Remarkably, among the subjects with secondary reactions 5 of 12 showed an anti– DeP4 response. Reactions with three or four antigens were not observed.

## Discussion

The detection of serotype-specific anti-DENV antibodies can be of value to characterize the complex anti-DENV immunity not only in individuals but also in larger groups in endemic areas. DENV type-specificity can be assessed by methods like RT-PCR or PRNT. However, this is only possible in samples taken either early (RT-PCR) or several months after infection (PRNT) to avoid cross-reactions. Therefore we looked for a simple but reliable alternative, which would allow the detection of DENV serotype-specific antibodies in samples of both patients and healthy subjects.

As we have shown here, the ICB ELISA, using four POD-labeled EDIII proteins (DeP1-4) as antigens, could reliably detect DENV serotype-specific antibodies in reconvalsescent patients with primary infection. Our data indicates, that serotype- specific IgG antibodies, reacting with DeP antigens and simultaneously with CD32, are detected only approx. two weeks after onset of fever, but obviously persist for several years, similar to DENV neutralizing antibodies. Possibly low avidity antibodies, produced shortly after onset of the disease, are not detected using the ICB ELISA, since CD32 preferentially binds high avidity antigen-antibody complexes [45]. The delayed antibody detection may partially explain the relatively low sensitivity (55 of 64 cases were detected) with samples taken shortly after onset of the disease. In acute infections a sensitivity of 87% to 93**%** was obtained by combining an NS-1 antigen test with IgM and IgG detection [50], [51]. Using various EDIII constructs as antigens for IgG or IgM antibody detection [23] a sensitivity of about 80% and specificity of almost 100% was obtained [34]. The small number of epitopes on the EDIII protein may explain why anti- EDIII antibodies are generally present at low levels in human immune sera [23].

In contrast to publications using EDIII proteins as antigens for the rapid diagnosis of acute DENV infections recent efforts were focused on the detection of DENV sero-type specific antibodies [37]. Midgley et al. [36] have performed competitive ELISAs, where multiple dilutions of serum and EDIII proteins were preincubated before the mixtures were applied to EDIII coated microtiter plates. Their data convincingly show that during primary DENV infection the maximum activity is directed against the homologous EDIII antigen of the infecting serotype. Our data on primary infections are in full agreement with their findings. However, using the ICB ELISA we found less cross-reactivity in our samples of patients with primary infection. Without competition only 15 of the 55 sera of our patients reacted with heterologous EDIII antigens and the cross-reactivity mainly confined to DeP1 and DeP3 antigens. The reason for this difference is unclear but may be due to different strategies used for both ELISAs. In contrast to indirect ELISAs the ICB ELISA preferentially detects high avidity antibodies via CD32 binding. Also the EDIII proteins bound to aggregated POD molecules may show an altered antigenicity. Finally, the presence of iTBEP in the ICB ELISAs, to avoid background reactions, may play a role.

On the EDIII domain, type-specific, subcomplex-specific and even a flavivirus-cross-reacting epitope have been recognized. The type-specific epitopes are located on the lateral ridge of the EDIII, as had been shown using mouse monoclonal antibodies [29], [31]. The flavivirus cross-reacting epitope, residing at the AB loop of EDIII, is predicted to have limited accessibility on the mature virus [29]. This may partially explain, why flavivirus cross-reactivity was not observed in our earlier investigations [41].

The interpretation of the ICB assay may be more difficult with samples of subjects with secondary DENV infection and due to repeated stimulation of the immune system antibodies to cross-reacting epitopes may play a major role. Thus, for studies in DENV-endemic countries with many secondary infections the competitive variant of ICB-ELISA should be applied, using the addition of heterologous EDIII (De1-4) antigens to eliminate cross-reacting antibodies directed to the subtype-specific epitopes on the DePs. As CD32 detects human and mouse antibodies equally well [41], [45] mouse monoclonal antibodies to subtype-specific EDIII epitopes might help to exclude residual cross-reactivities of the competitive ICB assays.

The low background reactions seen with the ICB ELISA depend on the use of highly labeled DeP antigens, which could be used at dilutions of 1∶≥16000. The high dilution was essential for the competitive assays, because the competitor proteins had to be added at a rather low dilution of only 1∶160 (100–200 -fold excess). However, due to the strong labeling of the antigens non-specific antibodies to *E. coli* proteins, His-tags or even the POD itself may occasionally be detected [41]. These non-specific reactions were suppressed successfully by adding similarly labeled but enzymatically inactive EDIII proteins such as iTBEP. In the presence of non-specific antibodies the addition of competitive antigens like De1-4 or iTBEP may occasionally result in large amounts of unlabeled immune complexes competing with the labeled ones for CD32. To avoid blocking of CD32 by unlabeled immune complexes, a high concentration of CD32 (5 µg/mL) was applied to the solid-phase. Therefore we had to start our own production of recombinant human CD32 to test hundreds of samples using four antigens per sample both with and without competition.

The results on serum samples collected in Vietnam in 1999 suggest that all four DENV serotypes have been circulating in that area for many years. Reports on the presence of all four DENV serotypes in Vietnam [42], [43], [52], [53] support our findings but the low proportion of only 21% secondary infections is in contrast to earlier data obtained with PRNT on the presence of secondary infections in Thailand [53], [54]}. Accordingly, our preliminary results obtained with samples of healthy Vietnamese subjects need further confirmation. In particular, a strong production of subtype-specific antibodies during DENV co-infections or subclinical infections [5], [43] has to be taken into account. The data of Midgley et al [36] suggest that during secondary DENV infection no type-specific antibodies to the new serotype can be detected, while only the primary type-specific and subtype-specific response is stimulated. Therefore several DENV infections in the Vietnamese subjects may have been misinterpreted as primary infections, when only the anamnestic response was detectable. However, we could identify two different type-specific responses in 21% of the DENV infected Vietnamese subjects. The age of our subjects may have played a role, who in contrast to children studied by Midgley et al. had a greater chance to experience numerous DENV infections, which may eventually trigger additional type-specific antibodies.

To prove the existence of different DENV type-specific responses in humans, additional adult patients with secondary DENV infection have to be studied. Patients showing a positive RT-PCR and containing high IgG antibody levels but no IgM antibodies to DENV [8], [49] are highly indicative of secondary infection. Follow-up samples of such patients can possibly be obtained during investigations in hyper-endemic areas.

The ICB ELISA can be applied for high throughput testing, because only a single incubation step is required. The method may help to extend our knowledge of the epidemiology of DENV infections in tropical areas.

## Supporting Information

Checklist S1 STARD Checklist.
(PDF)

Figure S1 TG-ROC analysis [55] of the four ICB ELISAs.
(PDF)

Figure S2 Antibody reactions (P/N ratios) of 55 patients using the competitive ICB ELISA with all DeP antigens.
(PDF)

Flowchart S1 The procedure for the collection of the serum samples of patients with DENV infection and of healthy subjects without DENV infection are shown.
(PDF)

Table S1 Intra- and inter-assay variation of DENV EDIII 1-4 ICB ELISAs.
(PDF)

Table S2 OD values used for figure 2.
(PDF)

Table S3 Antibody kinetics of four patients with DENV-1, -2, or -3 infection. Shown are the OD values used for figure 4.
(PDF)

Table S4 P/N data for 12 Vietnamese subjects with supposed secondary DENV infections.
(PDF)

## References

[1] Gubler DJ (1998) The global pandemic of dengue/dengue haemorrhagic fever: current status and prospects for the future. Ann Acad Med Singapore 27: 227–234. 9663316

[2] Shepard DS, Undurraga EA, Halasa YA (2013) Economic and disease burden of dengue in southeast Asia. PLoS Negl Trop Dis 7: e2055. 23437406 10.1371/journal.pntd.0002055 PMC3578748

[3] Halstead SB (1989) Antibody, macrophages, dengue virus infection, shock, and hemorrhage: a pathogenetic cascade. Rev Infect Dis 11 Suppl 4: S830–839. 2665015 10.1093/clinids/11.supplement_4.s830

[4] Halstead SB (2003) Neutralization and antibody-dependent enhancement of dengue viruses. Adv Virus Res 60: 421–467. 14689700 10.1016/s0065-3527(03)60011-4

[5] Chinnawirotpisan P, Mammen MP Jr, Nisalak A, Thaisomboonsuk B, Narupiti S, et al (2008) Detection of concurrent infection with multiple dengue virus serotypes in Thai children by ELISA and nested RT-PCR assay. Arch Virol 153: 2225–2232. 19011729 10.1007/s00705-008-0249-9

[6] Lang J (2009) Recent progress on sanofi pasteur's dengue vaccine candidate. J Clin Virol 46 Suppl 2: S20–24. 19800562 10.1016/S1386-6532(09)70291-4

[7] Durbin AP, Whitehead SS (2011) Next-generation dengue vaccines: novel strategies currently under development. Viruses 3: 1800–1814. 22069516 10.3390/v3101800 PMC3205382

[8] Laue T, Emmerich P, Schmitz H (1999) Detection of dengue virus RNA in patients after primary or secondary dengue infection by using the TaqMan automated amplification system. J Clin Microbiol 37: 2543–2547. 10405398 10.1128/jcm.37.8.2543-2547.1999 PMC85278

[9] Johnson BW, Russell BJ, Lanciotti RS (2005) Serotype-specific detection of dengue viruses in a fourplex real-time reverse transcriptase PCR assay. J Clin Microbiol 43: 4977–4983. 16207951 10.1128/JCM.43.10.4977-4983.2005 PMC1248506

[10] Chien LJ, Liao TL, Shu PY, Huang JH, Gubler DJ, et al (2006) Development of real-time reverse transcriptase PCR assays to detect and serotype dengue viruses. J Clin Microbiol 44: 1295–1304. 16597854 10.1128/JCM.44.4.1295-1304.2006 PMC1448645

[11] Chao DY, Davis BS, Chang GJ (2007) Development of multiplex real-time reverse transcriptase PCR assays for detecting eight medically important flaviviruses in mosquitoes. J Clin Microbiol 45: 584–589. 17108075 10.1128/JCM.00842-06 PMC1829073

[12] Qiu LW, Di B, Wen K, Wang XS, Liang WH, et al (2009) Development of an antigen capture immunoassay based on monoclonal antibodies specific for dengue virus serotype 2 nonstructural protein 1 for early and rapid identification of dengue virus serotype 2 infections. Clin Vaccine Immunol 16: 88–95. 19020106 10.1128/CVI.00212-08 PMC2620663

[13] Duong V, Ly S, Lorn Try P, Tuiskunen A, Ong S, et al (2011) Clinical and virological factors influencing the performance of a NS1 antigen-capture assay and potential use as a marker of dengue disease severity. PLoS Negl Trop Dis 5: e1244. 21811645 10.1371/journal.pntd.0001244 PMC3139664

[14] Kraus AA, Messer W, Haymore LB, de Silva AM (2007) Comparison of plaque- and flow cytometry-based methods for measuring dengue virus neutralization. J Clin Microbiol 45: 3777–3780. 17804661 10.1128/JCM.00827-07 PMC2168473

[15] Chan KR, Zhang SL, Tan HC, Chan YK, Chow A, et al (2011) Ligation of Fc gamma receptor IIB inhibits antibody-dependent enhancement of dengue virus infection. Proc Natl Acad Sci U S A 108: 12479–12484. 21746897 10.1073/pnas.1106568108 PMC3145677

[16] Shanaka WW, Rodrigo I, Alcena DC, Rose RC, Jin X, et al (2009) An automated Dengue virus microneutralization plaque assay performed in human Fc{gamma} receptor-expressing CV-1 cells. Am J Trop Med Hyg 80: 61–65. 19141841

[17] Thomas SJ, Nisalak A, Anderson KB, Libraty DH, Kalayanarooj S, et al (2009) Dengue plaque reduction neutralization test (PRNT) in primary and secondary dengue virus infections: How alterations in assay conditions impact performance. Am J Trop Med Hyg 81: 825–833. 19861618 10.4269/ajtmh.2009.08-0625 PMC2835862

[18] Heinz FX, Berger R, Tuma W, Kunz C (1983) Location of immunodominant antigenic determinants on fragments of the tick-borne encephalitis virus glycoprotein: evidence for two different mechanisms by which antibodies mediate neutralization and hemagglutination inhibition. Virology 130: 485–501. 6196909 10.1016/0042-6822(83)90102-2

[19] Roehrig JT (2003) Antigenic structure of flavivirus proteins. Adv Virus Res 59: 141–175. 14696329 10.1016/s0065-3527(03)59005-4

[20] Crill WD, Roehrig JT (2001) Monoclonal antibodies that bind to domain III of dengue virus E glycoprotein are the most efficient blockers of virus adsorption to Vero cells. J Virol 75: 7769–7773. 11462053 10.1128/JVI.75.16.7769-7773.2001 PMC115016

[21] Diamond MS, Pierson TC, Fremont DH (2008) The structural immunology of antibody protection against West Nile virus. Immunol Rev 225: 212–225. 18837784 10.1111/j.1600-065X.2008.00676.x PMC2646609

[22] Crill WD, Chang GJ (2004) Localization and characterization of flavivirus envelope glycoprotein cross-reactive epitopes. J Virol 78: 13975–13986. 15564505 10.1128/JVI.78.24.13975-13986.2004 PMC533943

[23] Wahala WM, Silva AM (2011) The human antibody response to dengue virus infection. Viruses 3: 2374–2395. 22355444 10.3390/v3122374 PMC3280510

[24] Lin HE, Tsai WY, Liu IJ, Li PC, Liao MY, et al (2012) Analysis of epitopes on dengue virus envelope protein recognized by monoclonal antibodies and polyclonal human sera by a high throughput assay. PLoS Negl Trop Dis 6: e1447. 22235356 10.1371/journal.pntd.0001447 PMC3250511

[25] Li PC, Liao MY, Cheng PC, Liang JJ, Liu IJ, et al (2012) Development of a humanized antibody with high therapeutic potential against dengue virus type 2. PLoS Negl Trop Dis 6: e1636. 22563515 10.1371/journal.pntd.0001636 PMC3341331

[26] Wahala WM, Donaldson EF, de Alwis R, Accavitti-Loper MA, Baric RS, et al (2010) Natural strain variation and antibody neutralization of dengue serotype 3 viruses. PLoS Pathog 6: e1000821. 20333252 10.1371/journal.ppat.1000821 PMC2841629

[27] Wahala WM, Kraus AA, Haymore LB, Accavitti-Loper MA, de Silva AM (2009) Dengue virus neutralization by human immune sera: role of envelope protein domain III-reactive antibody. Virology 392: 103–113. 19631955 10.1016/j.virol.2009.06.037 PMC2746956

[28] Williams KL, Wahala WM, Orozco S, de Silva AM, Harris E (2012) Antibodies targeting dengue virus envelope domain III are not required for serotype-specific protection or prevention of enhancement in vivo. Virology 429: 12–20. 22537810 10.1016/j.virol.2012.03.003 PMC3683589

[29] Sukupolvi-Petty S, Austin SK, Purtha WE, Oliphant T, Nybakken GE, et al (2007) Type- and subcomplex-specific neutralizing antibodies against domain III of dengue virus type 2 envelope protein recognize adjacent epitopes. J Virol 81: 12816–12826. 17881453 10.1128/JVI.00432-07 PMC2169112

[30] Matsui K, Gromowski GD, Li L, Schuh AJ, Lee JC, et al (2009) Characterization of dengue complex-reactive epitopes on dengue 3 virus envelope protein domain III. Virology 384: 16–20. 19101005 10.1016/j.virol.2008.11.013

[31] Brien JD, Austin SK, Sukupolvi-Petty S, O'Brien KM, Johnson S, et al (2010) Genotype-specific neutralization and protection by antibodies against dengue virus type 3. J Virol 84: 10630–10643. 20702644 10.1128/JVI.01190-10 PMC2950583

[32] Austin SK, Dowd KA, Shrestha B, Nelson CA, Edeling MA, et al (2012) Structural basis of differential neutralization of DENV-1 genotypes by an antibody that recognizes a cryptic epitope. PLoS Pathog 8: e1002930. 23055922 10.1371/journal.ppat.1002930 PMC3464233

[33] Simmons M, Nelson WM, Wu SJ, Hayes CG (1998) Evaluation of the protective efficacy of a recombinant dengue envelope B domain fusion protein against dengue 2 virus infection in mice. Am J Trop Med Hyg 58: 655–662. 9598457 10.4269/ajtmh.1998.58.655

[34] Batra G, Nemani SK, Tyagi P, Swaminathan S, Khanna N (2011) Evaluation of envelope domain III-based single chimeric tetravalent antigen and monovalent antigen mixtures for the detection of anti-dengue antibodies in human sera. BMC Infect Dis 11: 64. 21401963 10.1186/1471-2334-11-64 PMC3068959

[35] de Alwis R, Beltramello M, Messer WB, Sukupolvi-Petty S, Wahala WM, et al (2011) In-depth analysis of the antibody response of individuals exposed to primary dengue virus infection. PLoS Negl Trop Dis 5: e1188. 21713020 10.1371/journal.pntd.0001188 PMC3119640

[36] Midgley CM, Bajwa-Joseph M, Vasanawathana S, Limpitikul W, Wills B, et al (2011) An in-depth analysis of original antigenic sin in dengue virus infection. J Virol 85: 410–421. 20980526 10.1128/JVI.01826-10 PMC3014204

[37] Zidane N, Dussart P, Bremand L, Bedouelle H (2013) Cross-reactivities between human IgMs and the four serotypes of dengue virus as probed with artificial homodimers of domain-III from the envelope proteins. BMC Infect Dis 13: 302. 23815496 10.1186/1471-2334-13-302 PMC3701519

[38] Ludolfs D, Schilling S, Altenschmidt J, Schmitz H (2002) Serological differentiation of infections with dengue virus serotypes 1 to 4 by using recombinant antigens. J Clin Microbiol 40: 4317–4320. 12409419 10.1128/JCM.40.11.4317-4320.2002 PMC139635

[39] Ludolfs D, Niedrig M, Paweska JT, Schmitz H (2007) Reverse ELISA for the detection of anti West Nile virus IgG antibodies in humans. Eur J Clin Microbiol Infect Dis 26: 467–473. 17554572 10.1007/s10096-007-0309-1

[40] Ludolfs D, Reinholz M, Schmitz H (2009) Highly specific detection of antibodies to tick-borne encephalitis (TBE) virus in humans using a domain III antigen and a sensitive immune complex (IC) ELISA. J Clin Virol 45: 125–128. 19409841 10.1016/j.jcv.2009.03.016

[41] Schmitz H, Gabriel M, Emmerich P (2011) Specific detection of antibodies to different flaviviruses using a new immune complex ELISA. Med Microbiol Immunol 200: 233–239. 21533786 10.1007/s00430-011-0195-0

[42] Thai KT, Cazelles B, Nguyen NV, Vo LT, Boni MF, et al (2010) Dengue dynamics in Binh Thuan province, southern Vietnam: periodicity, synchronicity and climate variability. PLoS Negl Trop Dis 4: e747. 20644621 10.1371/journal.pntd.0000747 PMC2903474

[43] Coudeville L, Garnett GP (2012) Transmission dynamics of the four dengue serotypes in southern Vietnam and the potential impact of vaccination. PLoS One 7: e51244. 23251466 10.1371/journal.pone.0051244 PMC3519629

[44] Blessmann J, Van Linh P, Nu PA, Thi HD, Muller-Myhsok B, et al (2002) Epidemiology of amebiasis in a region of high incidence of amebic liver abscess in central Vietnam. Am J Trop Med Hyg 66: 578–583. 12201594 10.4269/ajtmh.2002.66.578

[45] Bruhns P, Iannascoli B, England P, Mancardi DA, Fernandez N, et al (2009) Specificity and affinity of human Fcgamma receptors and their polymorphic variants for human IgG subclasses. Blood 113: 3716–3725. 19018092 10.1182/blood-2008-09-179754

[46] Clos J, Brandau S (1994) pJC20 and pJC40–two high-copy-number vectors for T7 RNA polymerase-dependent expression of recombinant genes in Escherichia coli. Protein Expr Purif 5: 133–137. 8054844 10.1006/prep.1994.1020

[47] Mika A, Reynolds SL, Mohlin FC, Willis C, Swe PM, et al (2012) Novel scabies mite serpins inhibit the three pathways of the human complement system. PLoS One 7: e40489. 22792350 10.1371/journal.pone.0040489 PMC3394726

[48] Ortiz de Montellano PR, David SK, Ator MA, Tew D (1988) Mechanism-based inactivation of horseradish peroxidase by sodium azide. Formation of meso-azidoprotoporphyrin IX. Biochemistry 27: 5470–5476. 3179265 10.1021/bi00415a013

[49] Schilling S, Ludolfs D, Van An L, Schmitz H (2004) Laboratory diagnosis of primary and secondary dengue infection. J Clin Virol 31: 179–184. 15465409 10.1016/j.jcv.2004.03.020

[50] Pan-ngum W, Blacksell SD, Lubell Y, Pukrittayakamee S, Bailey MS, et al (2013) Estimating the true accuracy of diagnostic tests for dengue infection using bayesian latent class models. PLoS One 8: e50765. 23349667 10.1371/journal.pone.0050765 PMC3548900

[51] Fry SR, Meyer M, Semple MG, Simmons CP, Sekaran SD, et al (2011) The diagnostic sensitivity of dengue rapid test assays is significantly enhanced by using a combined antigen and antibody testing approach. PLoS Negl Trop Dis 5: e1199. 21713023 10.1371/journal.pntd.0001199 PMC3119643

[52] Pongsiri P, Themboonlers A, Poovorawan Y (2012) Changing pattern of dengue virus serotypes in Thailand between 2004 and 2010. J Health Popul Nutr 30: 366–370. 23082638 10.3329/jhpn.v30i3.12300 PMC3489952

[53] Tricou V, Minh NN, Farrar J, Tran HT, Simmons CP (2011) Kinetics of viremia and NS1 antigenemia are shaped by immune status and virus serotype in adults with dengue. PLoS Negl Trop Dis 5: e1309. 21909448 10.1371/journal.pntd.0001309 PMC3167785

[54] Wilder-Smith A, Yoksan S, Earnest A, Subramaniam R, Paton NI (2005) Serological evidence for the co-circulation of multiple dengue virus serotypes in Singapore. Epidemiol Infect 133: 667–671. 16050512 10.1017/s0950268805003821 PMC2870294

[55] Greiner M, Sohr D, Gobel P (1995) A modified ROC analysis for the selection of cut-off values and the definition of intermediate results of serodiagnostic tests. J Immunol Methods 185: 123–132. 7665894 10.1016/0022-1759(95)00121-p

