# Supplementary material for: Detection of Serotype-Specific Antibodies to the Four Dengue Viruses Using an Immune Complex Binding (ICB) ELISA
Source: PLoS Negl Trop Dis. 2013 Dec 26;7(12):e2580. doi: 10.1371/journal.pntd.0002580 (PMC3873247; doi:10.1371/journal.pntd.0002580)
Supplement: Figure S1 — TG-ROC analysis [55] of the four ICB ELISAs. (PDF) [file pntd.0002580.s002.pdf]

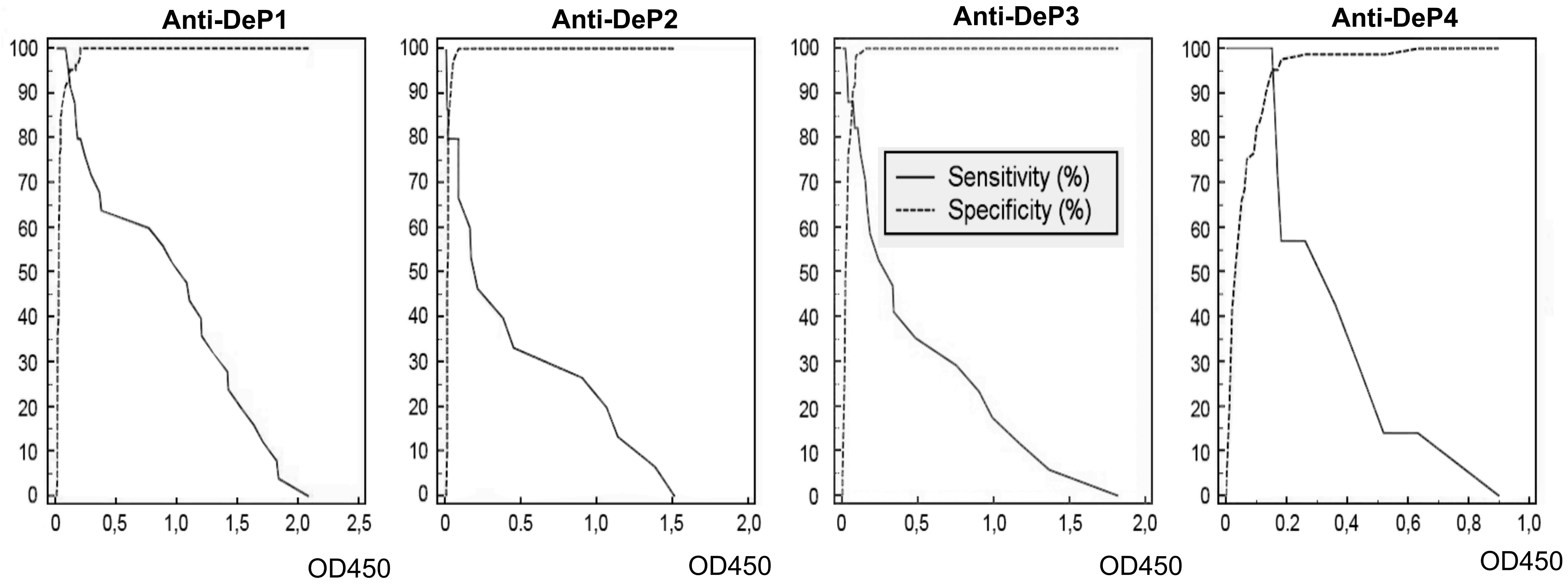

	Anti-DeP1	Anti-DeP2	Anti-DeP3	Anti-DeP4
**Cut-off**:	0.1:	0.09	0.1	0.16
**Sensitivity:**	96% (95%CI: 80-100%)	80% (95%CI: 64- 96%)	77% (95% CI: 50- 93%)	100% (95%CI: 35- 97%)
**Specificity**:	95% (95%CI: 88- 99%)	100 % (95%CI :96-100%)	99% (95% CI: 95-100%)	95% (95%CI: 89-99%)).

Figure S1.

TG-ROC analysis of the four ICB ELISAs. The OD values of 25 DENV-1, 15 DENV-2, 17 DENV-3, 7 DENV-4 samples were marked as positive (criterion 1) and the OD values of 110 antibody negative samples were marked as negative (criterion 0). Sensitivities and specificities are plotted against threshold (cut-off). Optimum cut-off values for each test can be determined. Sensitivities and specificities using cut-off values based on mean OD + 3σ of the 88 blood donor samples were also calculated. For all determinations, the area under curve (AUC) was >0.86 ($p < 0.0001$). AUC of 0.8-1 means good to excellent test performance (accuracy).
