# Supplementary material for: Detection of Serotype-Specific Antibodies to the Four Dengue Viruses Using an Immune Complex Binding (ICB) ELISA
Source: PLoS Negl Trop Dis. 2013 Dec 26;7(12):e2580. doi: 10.1371/journal.pntd.0002580 (PMC3873247; doi:10.1371/journal.pntd.0002580)
Supplement: Figure S2 — Antibody reactions (P/N ratios) of 55 patients using the competitive ICB ELISA with all DeP antigens. (PDF) [file pntd.0002580.s003.pdf]

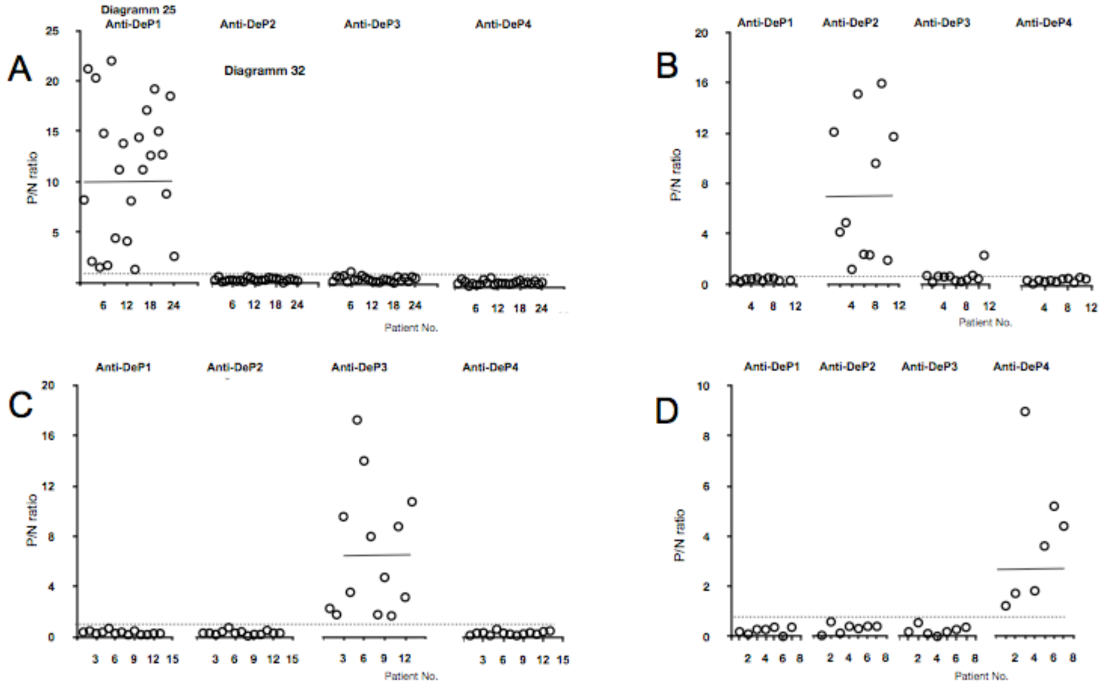

Figure S2

Figure S2. Antibody reactions (P/N ratios) of 55 patients using the ICB ELISA with all four DeP antigens. Samples of 24 patients with DENV-1 infection (A) and samples of 13 patients with DENV-3 infection (C) were tested with competition. Data in B (11 patients with DENV-2 infection) and D (7 patients with DENV-4 infection) were tested without competition and were taken from figure 2. P/N values were calculated using OD values/cut-off values.
