# Supplementary figures and images for: Detection of Serotype-Specific Antibodies to the Four Dengue Viruses Using an Immune Complex Binding (ICB) ELISA
Source: PLoS Negl Trop Dis. 2013 Dec 26;7(12):e2580. doi: 10.1371/journal.pntd.0002580 (PMC3873247; doi:10.1371/journal.pntd.0002580)

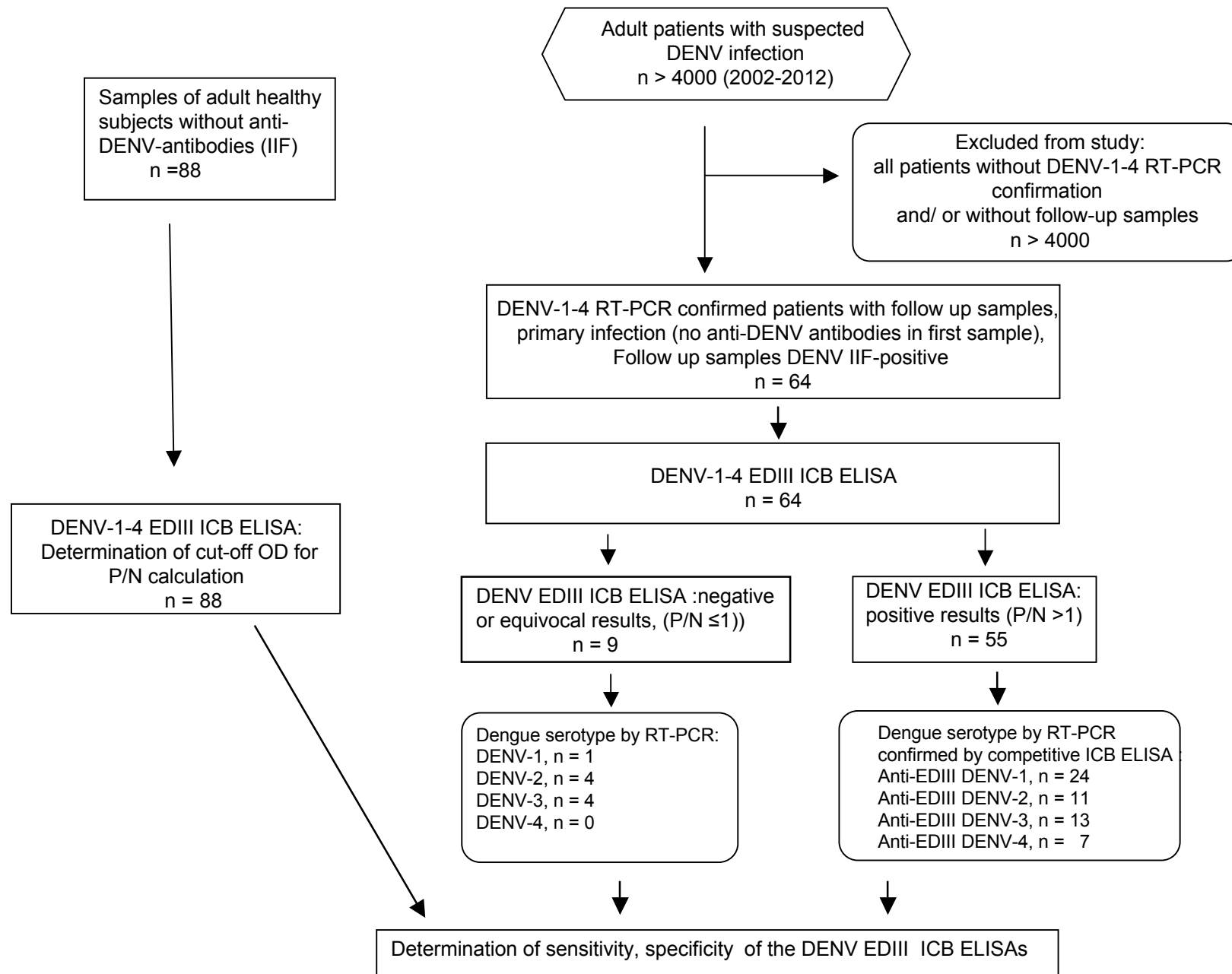

Supplement: Flowchart S1 — The procedure for the collection of the serum samples of patients with DENV infection and of healthy subjects without DENV infection are shown. (PDF) [file pntd.0002580.s004.pdf]
