# Supplementary material for: Detection of Serotype-Specific Antibodies to the Four Dengue Viruses Using an Immune Complex Binding (ICB) ELISA
Source: PLoS Negl Trop Dis. 2013 Dec 26;7(12):e2580. doi: 10.1371/journal.pntd.0002580 (PMC3873247; doi:10.1371/journal.pntd.0002580)
Supplement: Table S1 — Intra- and inter-assay variation of DENV EDIII 1-4 ICB ELISAs. (PDF) [file pntd.0002580.s005.pdf]

Table S1. Intra- and inter-assay variation of DENV EDIII 1-4 ICB ELISAs. Intra-assay CVs are calculated using six independent experiments measured in triplicates. Inter-assay CVs are calculated using four independent experiments measured in duplicates. Significant differences between negative and positive control were confirmed by standard t-test. Statistical analysis was performed using GraphPad Prism 6 Software (San Diego, USA). [1], calculated for P/N ratios.

		**Intra-assay**	**Inter-assay Evaluation**			
		**CV (%)**	**Abs mean ± SD**	**P/N ratio mean ± SD**	**CV[1] (%)**	**Significance**
**DENV1**	**Neg**	12.02	0.03 ± 0.002	0.34 ± 0.02	7.20	
	**Pos.**	5.19	0.82 ± 0.06	8.19 ± 0.62	7.56	p<0.001
**DENV2**	**Neg.**	9.48	0.03 ± 0.003	0.34 ± 0.03	8.82	
	**Pos.**	0.21	0.728 ± 0.04	7.3±0.4	5.3	p<0.001
**DENV3**	**Neg**	5.57	0.04 ± 0.004	0.35 ± 0.04	12.37	
	**Pos.**	8.54	1.06± 0.08	11.8 ± 0.92	7.8	p<0.001
**DENV4**	**Neg**	6.24	0.08 ± 0.006	0.53 ± 0.04	7.67	
	**Pos.**	2.01	0.81 ±0.033	5,08 ±0.21	4.1	p<0.001
