# Supplementary material for: Detection of Serotype-Specific Antibodies to the Four Dengue Viruses Using an Immune Complex Binding (ICB) ELISA
Source: PLoS Negl Trop Dis. 2013 Dec 26;7(12):e2580. doi: 10.1371/journal.pntd.0002580 (PMC3873247; doi:10.1371/journal.pntd.0002580)
Supplement: Table S2 — OD values used for figure 2. (PDF) [file pntd.0002580.s006.pdf]

Table S2. Antibody reactions of 55 dengue-positive sera to all four DENV DeP antigens using the ICB ELISA without competition. Shown are the OD values used for figure 2.

**DENV1** Patient No	DeP1	DeP2	DeP3	DeP4
1	0.85	0.04	0.03	0.12
2	2.02	0.25	0.34	0.14
3	0.2	0.02	0.09	0.28
4	2.04	0.03	0.62	0.05
5	0.17	0.04	0.03	0.06
6	1.57	0.04	0.33	0.05
7	0.18	0.03	0.08	0.08
8	2.32	0.26	1.13	0.34
9	0.42	0.02	0.23	0.10
10	1.07	0.23	0.11	0.37
11	1.33	0.01	0.25	0.07
12	0.41	0.05	0.08	0.08
13	0.72	0.12	0.03	0.08
14	0.13	0.05	0.03	0.14
15	1.34	0.05	0.11	0.11
16	1.22	0.23	0.24	0.08
17	1.69	0.05	0.06	0.10
18	1.20	0.06	0.42	0.42
19	1.81	0.05	0.32	0.09
20	1.58	0.01	0.03	0.11
21	1.44	0.21	0.42	0.06
22	0.98	0.1	0.03	0.12
23	1.90	0.03	0.51	0.07
24	0.27	0.03	0.08	0.10

**DENV2** Patient No	DeP1	DeP2	DeP3	DeP4
1	0.07	1.04	0.1	0.11
2	0.05	0.36	0.05	0.07
3	0.07	0.42	0.09	0.12
4	0.06	0.10	0.09	0.10
5	0.09	1.26	0.13	0.14
6	0.06	0.20	0.06	0.10
7	0.11	0.17	0.11	0.18
8	0.09	0.82	0.07	0.14
9	0.06	1.38	0.10	0.09
10	0.09	0.16	0.07	0.15
11	0.10	0.98	0.25	0.16

**DENV3** Patient No	DeP1	DeP2	DeP3	DeP4
1	0.05	0.04	0.24	0.05
2	0.05	0.04	0.15	0.05
3	0.06	0.02	0.90	0.08
4	0.04	0.04	0.34	0.06
**5**	0.71	0.30	1.82	0.07
6	0.15	0.03	1.36	0.08
7	0.24	0.05	0.75	0.06
8	0.06	0.01	0.18	0.06
9	0.09	0.02	0.48	0.06
10	0.03	0.02	0.16	0.06
11	0.15	0.03	0.99	0.07
12	0.02	0.03	0.33	0.08
13	0.04	0.03	1.17	0.34

**DENV4** Patient No	DeP1	DeP2	DeP3	DeP4
1	0.03	0.01	0.03	0.19
2	0.02	0.06	0.06	0.27
3	0.04	0.02	0.02	1.44
4	0.04	0.04	0.01	0.29
5	0.05	0.03	0.03	0.58
6	0.01	0.04	0.04	0.83
7	0.05	0.04	0.05	0.7
