# Supplementary material for: Detection of Serotype-Specific Antibodies to the Four Dengue Viruses Using an Immune Complex Binding (ICB) ELISA
Source: PLoS Negl Trop Dis. 2013 Dec 26;7(12):e2580. doi: 10.1371/journal.pntd.0002580 (PMC3873247; doi:10.1371/journal.pntd.0002580)
Supplement: Table S3 — Antibody kinetics of four patients with DENV-1, -2, or -3 infection. Shown are the OD values used for figure 4. (PDF) [file pntd.0002580.s007.pdf]

Table S3. Antibody kinetics of four patients with DENV-1, -2, or -3 infection. Shown are the OD values used for figure 4

Patient	Days	DeP1 +De3	DeP2 + De3	DeP3 +De1	DeP4 +De3	DeP1	DeP2	DeP3	DeP4
**A**	3	**0.04**	0.08	0.12	0.16	**0.04**	0.08	0.13	0.16
DEN-1	9	**1.81**	0.04	0.18	0.19	**1.70**	0.18	0.55	0.19
	26	**2.30**	0.04	0.25	0.26	**2.20**	0.05	0.91	0.21
**B**	14	0.03	**0.03**	0.04	0.03	0.03	**0.13**	0.06	0.20
DEN-2	25	0.03	**0.37**	0.05	0.17	0.03	**0.37**	0.07	0.18
	200	0.02	**1.61**	0.09	0.08	0.02	**1.48**	0.06	0.07
**C**	4	**0,11**	0,09	0.05	0.06				
DEN-1	8	**0.84**	0.5	0.09	0.17				
**D**	14	0.09	0.05	**0.31**	0.15				
DENV-3	**17**	0.05	0.04	**0.55**	0.13				
