# Supplementary material for: Detection of Serotype-Specific Antibodies to the Four Dengue Viruses Using an Immune Complex Binding (ICB) ELISA
Source: PLoS Negl Trop Dis. 2013 Dec 26;7(12):e2580. doi: 10.1371/journal.pntd.0002580 (PMC3873247; doi:10.1371/journal.pntd.0002580)
Supplement: Table S4 — P/N data for 12 Vietnamese subjects with supposed secondary DENV infections. (PDF) [file pntd.0002580.s008.pdf]

Table S4. P/N data for 12 Vietnamese subjects with supposed secondary DENV infections. Using the competitive ELISA results with two different DeP antigens were obtained (serum dilution 1:100).

Patient No	DeP1	DeP2	DeP3	DeP4	Serotype
26	**2.7**	0.2	0.7	**7.6**	1, 4
32	1.1	1.1	**8.4**	**9.2**	3, 4
44	**6.1**	0.5	**2.3**	0.8	1, 3
53	**8.2**	0.8	**6.7**	0.3	1, 3
59	0.8	0.7	**5.2**	**2.0**	3, 4
61	**3.6**	0.2	**4.5**	0.9	1, 3
69	**5.1**	0.5	**8.5**	0.8	1, 3
71	0.8	0.2	**10.3**	**6.5**	3, 4
75	**2.7**	**7.1**	0.8	0.5	1, 2
77	**2.1**	**5.3**	0.7	1.1	1, 2
81	**4.4**	0.9	0.9	**7.2**	1, 4
85	**2.6**	**8.5**	0.7	0.5	1, 2
